# Electron Microscopy Characterization of the High Temperature Degradation of the Aluminide Layer on Turbine Blades Made of a Nickel Superalloy

**DOI:** 10.3390/ma13143240

**Published:** 2020-07-21

**Authors:** Mariusz Bogdan, Witold Zieliński, Tomasz Płociński, Krzysztof Jan Kurzydłowski

**Affiliations:** 1 Department of Mechanical Engineering, Bialystok Technical University, 45 Wiejska, 15333 Białystok, Poland; k.kurzydlowski@pb.edu.pl; 2 Faculty of Materials Science and Engineering, Warsaw University of Technology, Woloska 141, 02507 Warszawa, Poland; Witold.Zielinski@pw.edu.pl (W.Z.); tomasz.plocinski@pw.edu.pl (T.P.)

**Keywords:** turbine blade, nickel-based superalloys, aluminide layer, high temperature, electron microscopy

## Abstract

The effects of exposure to overheating (temperature above 1000 °C) on the degradation (modification) of layers of coatings (coatings based on aluminum) of uncooled polycrystalline rotor blades of aircraft turbine jet engines were investigated under laboratory conditions. In order to determine the nature of the changes as well as the structural changes in the various zones, a multi-factor analysis of the layers of the coating, including the observation of the surface of the blades, using, among others, electron microscopy, structural tests, surface morphology, and chemical composition testing, was carried out. As a result of the possibility of strengthening the physical foundations of the non-destructive testing of blades, the undertaken research mainly focused on the characteristics of the changes occurring in the outermost layers of the coatings. The obtained results indicate the structural degradation of the coatings, particularly the unfavorable changes, become visible after heating to 1050 °C. The main, strongly interacting, negative phenomena include pore formation, external diffusion of Fe and Cr to the surface, and the formation and subsequent thickening of Fe-Cr particles on the surface of the alumina layer.

## 1. Introduction

Aircraft engine turbine rotor blades are exposed to very demanding in-service conditions such as, among others, varying mechanical aerodynamic/thermal loads and the generally high-temperature of exhaust gases. Among the above-mentioned blade degrading factors, a high service temperature is of special importance, as it rapidly accelerates the diffusional processes taking place within the blades. In order to reduce the temperature of the superalloy of the blades, a number of measures are taken, such as allowing for internal cooling and the application of coatings [1,2,3,4,5]. On the other hand, there is a strong tendency to improve the performance of aircraft engines by increasing the temperature of its exhaust gases. Nickel alloys, for example, are covered using aluminum-based coatings. The blades of an aircraft jet engine’s rotor are provided with a thermo-resistant layer (a diffusion coating) having a high resistance to oxidation and high-temperature corrosion. Aluminum diffusion coatings can be produced using, among others, the pack cementation, the out of pack, the slurry processes, or the chemical vapor deposition methods [6,7,8,9,10]. Aluminum coating utilizing CVD (Chemical Vapour Deposition), one of the most up to date techniques, allows for the covering of complex-shaped elements, both internal and external, thanks to which it has found use in coating turbine blades with cooling channels [3,4,5,11]. Diffusion aluminide coatings as bond coats in thermal barrier coating (TBC) systems have also been applied on turbine engine hot section blades and vane segments, as protection from the aggressive conditions of industrial and aero-gas turbines [12,13]. Among the several compounds of aluminum that can form during the coating process, the β-(NiAl) intermetallic phase turns out to be the best compromise, offering high-temperature resistance and a mix of mechanical properties. In order to raise the thermal stability of this phase composition of the coating’s material, it is augmented with additional materials: Pt, Ta, Hf, Y, Si, and others [14,15,16,17,18]. The addition of yttrium allows for the avoidance of porosity during exploitation, and increases the adhesion of the created oxides [19,20]. The rate of material oxidation, the basic mechanism of corrosion, grows exponentially along with the temperature. Utilized coatings improve thermo-resistance, which affects, among others, blade durability, impacting the life and dependability of the entire jet turbine. During exploitation, on the surface of the β-NiAl layer, a film of aluminum oxides form, Al_2_O_3_, which protect the material from further oxidation or, more precisely, significantly reduce the rate of oxidation [21,22,23]. If the created scale is impermeable and uniform, then it further protects from corrosion.

Under in-service conditions, the coated elements are exposed to high temperatures, which may change both the structure of the aluminide layer and the underlying substrate [24,25,26,27]. For obvious reasons, these changes are expected to be especially pronounced in the outermost part of coatings, which reach the highest temperatures and are exposed to direct contact with ambient gasses. On the other hand, in the near past, the direct investigation of these sections of coatings was nearly impossible, because of the difficulty in obtaining representative samples, as they extend only a few microns from the surface. At the same time, understanding the processes taking place in the coating during their exposure to high temperatures is of key importance in predicting the remaining time-of-life of the elements they are meant to protect, as well being essential to the development of Non-Destructive Testing (NDT) procedures for the evaluation of those components after they have been exposed to in-service conditions. One such procedure, based on the color analysis of the light reflected from the protective coating, has been put forward by M. Bogdan [28,29,30]. In order to strengthen the physical basis of this procedure, it is desirable to gain insight into the changes taking place in the outermost layers of the aluminide layer exposed to extreme heating. 

Within this context, the aim of the present study was to investigate the changes in the aluminum-based coatings of the superalloy exposed to temperatures exceeding 1000 °C. To this end, Scanning Electron Microscopy (SEM), high-resolution Scanning Transmission Electron Microscopy (STEM), and Transmission Electron Microscopy (TEM) have been used in combination with Focus Ion Beam (FIB) sample preparation. As one of the more general aims of this study was to correlate the changes in the structure of the aluminide layer with their appearance, special attention was paid to the outermost part of the coatings.

## 2. Materials and Methods 

The jet engine rotor blades used for the aims of the present study were all cast at the same time. In the initial phase of the process, bars of EI-867 alloy (developed in Russia) were non-invasively inspected for micro-cracks using ultrasound, followed by cutting out the blades to pre-specified dimensions and weight. The next stage consisted of preliminary forging and finishing the forging to the dimensions from the blueprint. The created elements’ blades and hubs were then checked with control instruments with respect to their final dimensions. After that, the parts were placed in a muffle for heat-treating, supersaturation, and aging. The process of supersaturation was carried out at temperatures ranging from 1200 to 1220 °C for 4–6 h, and cooling occurred in the air. Aging occurred at a temperature of 950 °C and lasted for 7–10 h, with subsequent cooling in air. Cooling in the air during the supersaturation leads to the emission of very small γ’ phase particles, whose relative volume is approximately 20%. Aging causes the further emission of γ’ phase particles and the growth of existing ones. Long-lasting, additional subjection to high temperatures changes their content—from 32% (800 °C) to about 8% (1100 °C). Subsequently, the elements were sandblasted and subjected to thermochemical treatment (the process of aluminizing). The aluminizing was realized using the out of pack method at a temperature of 950 °C and for a duration of 12 h.

The blades coated with such aluminide coatings were exposed for 1 h to temperatures ranging from 950 °C to 1150 °C in a vacuum obtained utilizing a TURBOVAC 50 vacuum pump. Temperatures reaching 900–950 °C were recommended as the maximum allowable temperatures [28], because it is likely that they may be reached in emergency conditions during aircraft jet engine operation. 

The considered aluminum-based coatings were applied to the superalloy with a chemical composition given in Table 1. The alloy’s structure is typical of nickel superalloys for plastic forming and consists of a γ phase, a γ’ phase, and carbides. The γ phase is a solid solution of chromium, cobalt, molybdenum, and tungsten in nickel. The γ’ (Ni_3_Al) particles have a cubic shape. The blades made of this superalloy can work in temperatures reaching 900 °C. 

**Figure 1:**
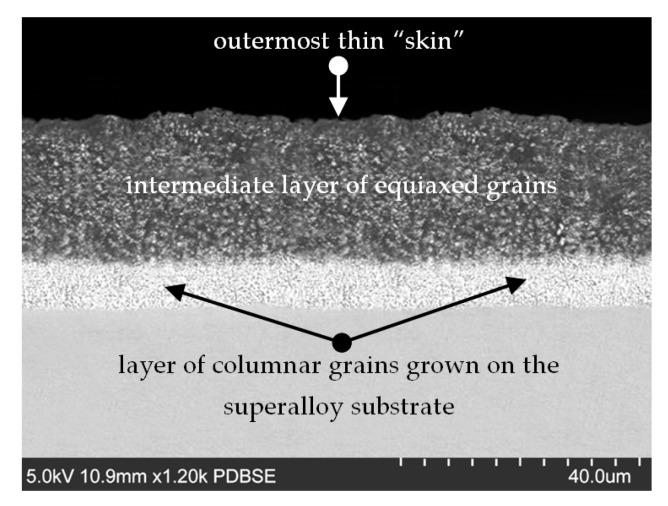
Example of an SEM image of a cross-section of the layer on as-coated blades prior to exposure to temperatures applied in this study.

A representative SEM image (SU-70, Hitachi, Japan) of a cross-section of the aluminide layer deposited on the blade made of the superalloy in question is shown in Figure 1. Taking into account the duration of the aviation tasks, the time of the exposure to the temperatures applied in the current study was 1 h.

Taking into account the duration of aviation tasks, the time of exposure to the temperatures applied in the current study was 1 h. The samples for SEM and STEM observations were prepared using the FIB technique (FIB 2100, Hitachi, Japan). Prior to cutting the samples out, the blades were coated with a protective layer of copper, and subsequently with a layer of tungsten. Coating with tungsten is a standard procedure used in the FIB technique. It is carried out in relatively low temperatures, and the W atoms do not migrate into the samples investigated. Additional copper coatings improve the SE images obtained with FIB. High-resolution STEM imaging was performed using a Hitachi HD2700, under an accelerating voltage of 200 kV, equipped with an EDS detector for chemical composition analyses (ThermoFisher, Noran Six, USA). 

## 3. Results

### 3.1. New Blades (Prior to the Exposure to High Temperatures)

A representative image of the cross-section of the coating layers deposited on the blades investigated in this study is shown in Figure 1. 

The analyzed coatings had a thickness of c.a. 28 μm. They consisted of three distinct sub-layers:

(a) The outermost thin “skin”
(b) An intermediate layer of equiaxed grains
(c) A layer of columnar grains grown on the superalloy substrate.

Such a combination for the sub-layer is typical of the coatings fabricated by the out of pack, and provides the required protection to the turbine blades investigated in this study.

### 3.2. Coatings on the Blades Exposed to 1050 °C

**Figure 2:**
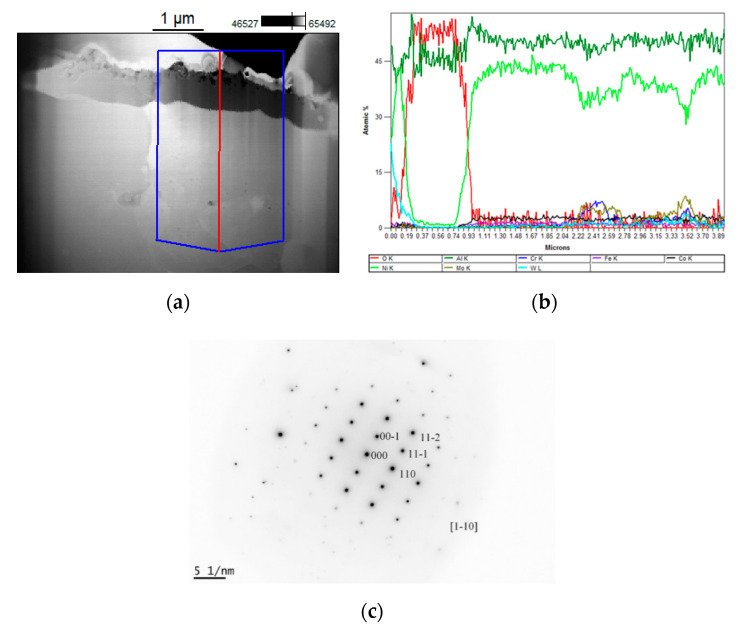
Microstructures, linear scans of the chemical composition, and phase identification representative of layers on the nickel superalloy: (**a**) STEM image of a cross-section; (**b**) linear scans of alloying elements; (**c**) Selected Area Diffraction (SAD) pattern of the NiAl disordered alloy.

SEM, STEM, and TEM (diffraction) studies revealed that after exposure to a temperature of 1050 °C, the outer most 1 μm skin consisted of alumina Al_2_O_3_. The layer below was made up of NiAl—see Figure 2.

The results reported earlier show that the coatings’ thickness nearly doubled and reached about 45 µm when the coated blades were exposed to temperatures exceeding 1000 °C for 1 h [25,28]. In fact, after exposure to 1000 °C for 1 h, the thickness of the coatings nearly doubled, reaching 45 μm. This increase in the thickness was accompanied by the degradation of the structure and properties of the coatings, and the substrate was some distance from the coating/superalloy interface [24,28].

### 3.3. Comparison of the Coatings Exposed to 1050 and 1150 °C

In order to obtain better in-sight into the processes taking place in the coatings after exposure to high temperatures, cross-sections of the coated blades after exposure to 1050 and 1150 °C for 1 h were investigated. The samples for the SEM investigations were prepared by ion cross section milling in a Hitachi IM400 system. The samples for the investigations with TEM/STEM were prepared busing the FIB lift out technique. The SEM observations were taken with a BSE detector at an accelerating voltage of 5 kV, where the atomic mass contrasts were dominating. 

**Figure 3:**
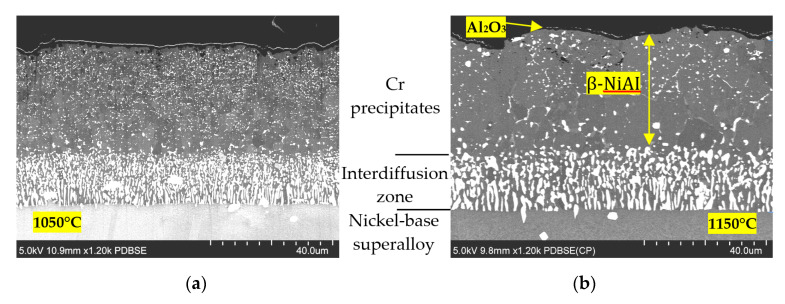
Comparison of the structure of the layers on the superalloy after exposure to temperatures of: (**a**) 1050 °C; (**b**) 1150 °C.

The results of the SEM observations are visualized in Figure 3. It can be noted that 1 h of exposure to these temperatures resulted in coating degradation, as manifested by the increasing number of pores. There was also visible tendency for microstructure coarsening, particularly noticeable in the case of the blades exposed to 1150 °C.

### 3.4. Surface Morphologies and Microstructure of Near-Surface Zone 

**Figure 4:**
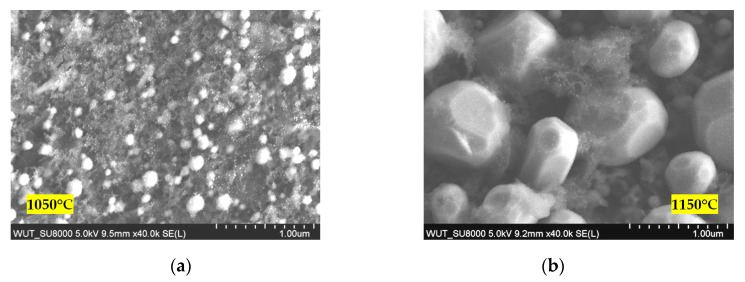
Morphology of the surface of the samples exposed to: (**a**) 1050 °C; (**b**) 1150 °C.

The morphologies of the surfaces of samples exposed to temperatures of 1050 and 1150 °C are shown in Figure 4.

**Figure 5:**
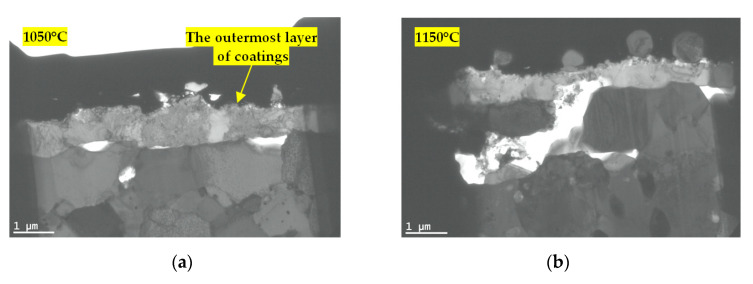
TEM images of the cross-section of the surface coatings on the blades exposed to: (**a**) 1050 °C; (**b**) 1150 °C.

The TEM images of cross-sections of blades exposed to the temperatures of 1050 and 1150 °C are shown in Figure 5.

**Figure 6:**
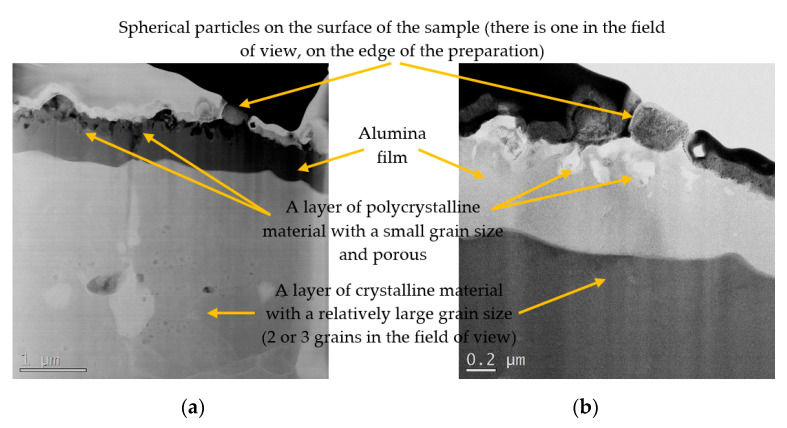
Cross-section of the coating exposed to 1150 °C, imaged with HR STEM, employing: (**a**) Z—contrast; (**b**) TE—contrast.

Figure 6 shows the porosity. It should be noted that it is highly localized in a narrow sub-layer underneath the outer surface. The thickness of this pore-rich layer is lower than 0.2 μm. Within this layer, the porosity is high, approaching c.a. 30%. However, the pores are sub-micron in size and their density is difficult to estimate experimentally.

The TEM observations of the samples exposed to 1050 and 1150 °C can be summarized in the following manner:

- the delamination of the coating layer was not observed in the study;
- the outer most layer of coatings, made of alumina, is approximately 1 μm thick;
- the size of alumina grains is much larger in the sample exposed to 1150 °C, and exceeds the thickness of the layer;
- there are numerous pores present in the coatings, which are much smaller in the case of the samples exposed to 1050 °C and are located underneath alumina layer,
- particles on the outer surface are observed, which, in the case of the exposure to 1050 °C, have an irregular shape, while in the case of 1150 °C, they are spherical.

**Figure 7:**
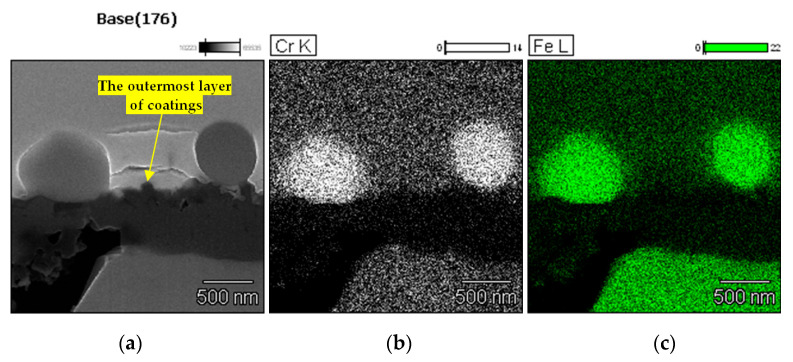
The microstructure and chemical composition of the particles revealed on the surface of the blades exposed to 1150 °C: (**a**) section selected for observations; (**b**) concentration of Cr; (**c**) concentration of Fe.

The chemical composition of the microstructural elements shown in the images presenting sections of blades exposed to 1150 °C was measured using EDS (ThermoFisher, Noran Six, USA). The results of these measurements relevant to the topic of this report are shown in Figure 7.

The results of the EDS measurements of the chemical composition show that the particles appearing on the surface of the blades exposed to 1050 and 1150 °C are made of Cr and Fe.

The diffusional processes taking place in the coatings under exposure to the temperatures applied in the present study can be explained based on the results of the work by Pedraza et al. [31], who investigated both the isothermal and cyclic oxidation of aluminum-rich coatings. The current paper is complementary to [31] because it focuses on the very top near outer layer of the coating. The current paper demonstrates that with the exception of the formation of the alumina layer, all other occurring processes such as coarsening of the microstructure of the coatings, formation of pores, outward diffusion of Fe and Cr to the surface, degrade the insulation and protective properties of the heat-resistant coatings. The processes of the microstructure coarsening and the formation of pores have been reported in the past, as seen in, for example [25,30]. Two processes (outward diffusion of Fe and Cr to the surface, and the formation and subsequent coarsening of Fe-Cr particles on the surface of the alumina layer), to the best knowledge of the authors, have not so far received much attention. On the other hand, they are indicative of advanced stages of coating degradation, and as such, are important in the context of evaluating the possibility for their further exploitation. The diffusion of Cr and Fe and the formation of the Cr/Fe rich particles on the surface of the alumina take place at the same stage of coating degradation as the formation of large pores. Under the experimental conditions employed in the current study, these phenomena are particularly advanced with respect to the blades exposed to 1150 °C. As has been stated earlier [28,29,30], this degradation is accompanied by changes in light reflection, which can be used for the non-destructive monitoring of the blades’ fitness to service. The results of the present study provide a firm microstructural basis for the utilization of the light reflection method, and contribute insight into the degradation processes of the blades.

## 4. Conclusions 

The basic requirement for an aluminide layer is connected with high temperature oxidation and the protection of turbine blades against hot corrosion. Acting as a protective coating, the layer is exposed to high temperatures, which may cause its structural degradation/modification. As a general rule, temperatures exceeding the temperatures applied during the coating are expected to induce diffusional processes, leading to phase transformations and to the redistribution of the alloying elements. These processes become particularly intensive when the temperature significantly exceeds 900 °C, which is recommended as the maximum operating temperature for blades made of the specified superalloy.

Based on the SEM/TEM investigations carried out in the present study, the following microstructural processes have been identified as taking place in the coatings exposed to temperatures exceeding 1000 °C in a vacuum of 9 mbar:

- formation of a thin layer of alumina Al_2_O_3_,
- coarsening of the microstructure of the coatings,
- formation of pores,
- outward diffusion of Fe and Cr to the surface,
- formation and subsequent coarsening of Fe-Cr particles on the surface of the alumina layer.

In the context of non-destructive inspection of the blades exposed to high temperatures, the last process among those listed above is of special meaning, as it explains the changes in the reflected light, already reported by [28,30].

## Figures and Tables

**Table 1:** Chemical composition of the superalloy used in the present study (wt.%).

C	Mn	Si	Cr	Fe	Co	Mo	W	Al	B	Ni
max	max	max							max	Rest
0.1	0.3	0.6	9.0	4.0	14	10.3	5.0	4.5	0.02	
